# Uncontrolled Acromegaly Resulting in the Need for Left Ventricular Assist Device as Bridge to Heart Transplant

**DOI:** 10.1210/jcemcr/luae072

**Published:** 2024-05-14

**Authors:** Megana Murugesh, Franklin Llereña Thelmo, Monika Khanna Shirodkar

**Affiliations:** Department of Internal Medicine, Thomas Jefferson University Hospital, Philadelphia, PA 19107, USA; Department of Endocrinology, Thomas Jefferson University Hospital, Philadelphia, PA 19107, USA; Department of Endocrinology, Thomas Jefferson University Hospital, Philadelphia, PA 19107, USA

**Keywords:** acromegaly, LVAD, valvular heart disease, heart transplant

## Abstract

Acromegaly is a rare pituitary condition stemming from hypersecretion of growth hormone (GH). Classic presentation involves enlarged hands, feet, and coarse facial features. However, late-onset cardiac manifestations develop in the absence of disease control. Of the various cardiac complications, heart failure is the rarest (3%-4% of cases). Here we present a case of acromegaly diagnosed after the patient exhibited symptoms of heart failure, with eventual placement of a left ventricular assist device (LVAD) as a bridge to orthotopic heart transplant. The 37-year-old patient originally presented with exercise intolerance and “heavy heartbeats” but was found to be in acute decompensated heart failure, with an ejection fraction (EF) of 15%. The acromegaly diagnosis was confirmed with labs, and he began treatment with lanreotide 120 mg weekly along with 0.5 mg cabergoline twice weekly. EF improved up to 30%. Soon after, he was lost to follow-up during the COVID-19 pandemic and returned with worsening EF. An LVAD was placed to support recovery while the patient awaited heart transplant. While LVADs are a common measure of cardiac support for ischemic cardiomyopathy, they can also be successful options in the setting of GH-driven cardiomegaly.

## Introduction

Acromegaly is a rare pituitary condition stemming from hypersecretion of growth hormone (GH), most commonly from a pituitary adenoma. The disease is exceedingly rare, with an incidence ranging from 3 to 11 persons per million per year and a prevalence of 60 persons per million per year. It is a systemic, progressive, and, if untreated, destructive disease associated with a spectrum of multi-organ comorbidities. Immediate treatment is crucial (1). Acromegaly classically presents with enlarging hands or feet, and development of coarse facial features. Cardiac complications are late-stage developments of untreated acromegaly, and classic heart failure on initial presentation is rare. We present a case of acromegaly diagnosed during initial presentation with heart failure, resulting in the need for a left ventricular assist device (LVAD) to support the patient's convalescence and tumor resection.

## Case Presentation

A previously healthy 37-year-old male presented to the emergency room with chest pain and shortness of breath. At presentation, the patient had no significant medical history: no known hypertension, diabetes, or other cardiovascular issues. Since he had not seen a physician in decades, there were no prior laboratory values. He frequently exercised. He never used cigarettes, alcohol, or recreational drugs. His family history was unremarkable. The patient noted a growth spurt between the ages of 16 and 18 years, attaining his current height of 1.96 meters. He also endorsed facial feature changes during this time—per patient, he looked taller and different from his family. On examination, the patient had a blood pressure of 102/70 mmHg, heart rate of 83 beats per minute, respiratory rate of 18 breaths per minute, oxygen saturation of 99% on room air, and temperature of 36.6 °C (97.9 °F). The patient had marked acromegalic facial features, including frontal bossing, macrognathia, and enlarged hands and feet. He denied headaches, vision changes, or excess sweating. The only symptoms he experienced were occasional heart palpitations and “heavy heartbeats.”

At an outside hospital, a transthoracic echocardiogram (TTE) showed an ejection fraction (EF) of 15%, suspicious for new acute cardiomyopathy. Cardiac MRI did not show perfusion defects or delayed gadolinium enhancement concerning for fibrosis. Endomyocardial biopsy showed no infiltrative disease but demonstrated moderate hypertrophy with mild fibrosis. Right heart catheterization showed elevated pressures. His initial insulin-like growth factor 1 (IGF-1) level was 455 ng/mL (59.61 nmol/L) (reference range: 52-328 ng/mL). Although a GH level was ordered and was documented as “in process,” the result is not available. Magnetic resonance imaging (MRI) confirmed the presence of a 10 mm unenhancing pituitary mass. At the time, conservative medical management was opted over trans-sphenoidal surgery (TSS) by the outside heart failure team due to increased intraoperative cardiovascular risk and morbidity due to adenoma proximity to vasculature. Surgery was deferred, and the patient was started on lanreotide injections and titrated up to a dose of 120 mg weekly, along with cabergoline 0.5 mg twice weekly. Over the next year, the patient had improvement in his left ventricular ejection fraction (LVEF) up to 25% to 30%, with IGF-1 levels slowly down-trending. His last known IGF-1 level was 260 ng/mL (34.06 nmol/L).

Unfortunately, the patient was lost to follow-up for 2 years due to the COVID-19 pandemic care shortages and loss of insurance causing inability to afford medications. There was no further known discussion at the outside hospital regarding the addition of pegvisomant for better biochemical control. He later presented to our sister institution with worsening heart failure requiring intravenous milrinone. The patient was transferred to our main institution for further multidisciplinary management.

## Diagnostic Assessment

**Figure 1.**
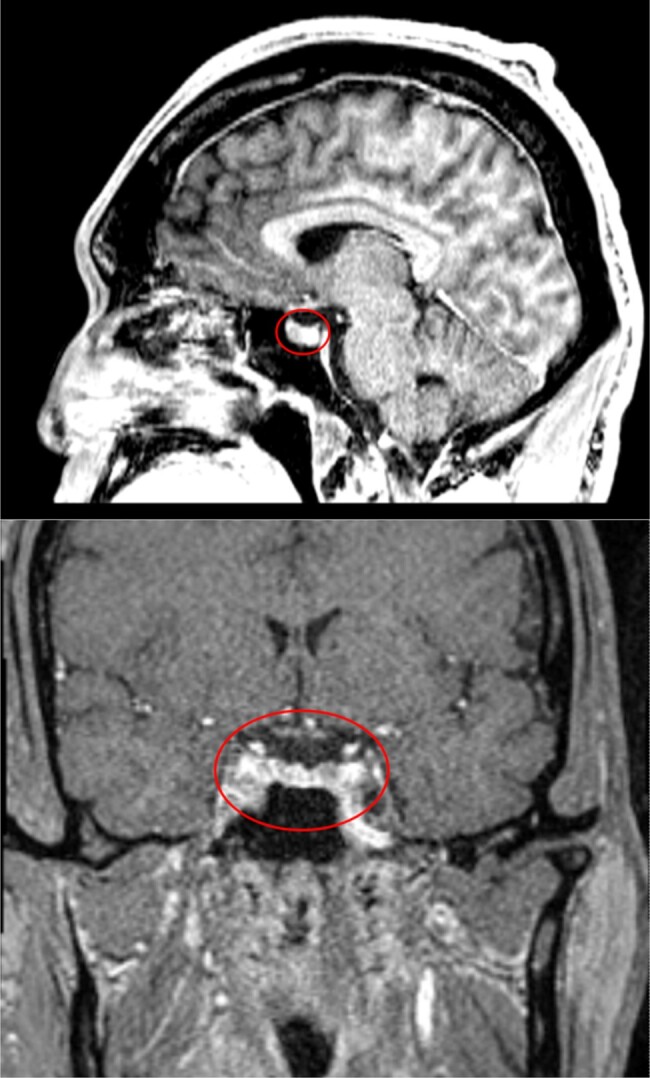
MRI of the pituitary (coronal and sagittal views) showing 10-mm macroadenoma, marked by red circle. Frontal bossing of cranium can be seen to the left of the sagittal image.

On arrival at our institution, the patient stated that he had not taken his acromegaly-controlling medication for at least 3 months. In February 2022, his GH was 14.8 ng/mL (14.8 µg/L), with an IGF-1 level of 270 ng/mL (35.37 nmol/L) (Table 1). Remaining pituitary workup revealed monomeric prolactin level of 14 µg/L (608.69 pmol/L) and a thyrotropin (TSH) level of 2.88 µU/mL. Adrenocorticotropic hormone (ACTH), follicle-stimulating hormone (FSH), and luteinizing hormone (LH) levels were not sent until later. Pituitary MRI revealed a 7.0 × 10.0 × 7.0 mm adenoma in the left anterior aspect of the pituitary gland with no optic chiasm involvement (Fig. 1). Repeat cardiac evaluation included TTE showing EF 15% with global hypokinesis.

**Table 1.** IGF-1 levels and Z scores before and after transsphenoidal surgery (TSS)

Pre/post TSS	IGF-1(reference range: 52-328 ng/mL)	Z-score	GH(reference range: < 10 ng/mL)
2 years pre-op	455 ng/mL (59.61 nmol/L)	2.7	n/a
8 months pre-op	270 ng/mL (35.37 nmol/L)	1.5	14.8 ng/mL (14.8 µg/L)
4 days pre-op	528 ng/mL (69.17 nmol/L)	3.2	4.2 ng/mL (4.2 µg/L)
2 days post op	396 ng/mL (51.88 nmol/L)	2.5	n/a
17 days post-op	308 ng/mL (40.35 nmol/L)	1.8	n/a
∼2 months post-op	297 ng/mL (38.91 nmol/L)	1.7	n/a
∼4 months post-op	248 ng/mL (32.49 nmol/L)	1.3	n/a
∼2 years post-op	240 ng/mL (31.37 nmol/L)	1.3	1.8 ng/mL (1.8 µg/L)

Growth hormone levels before and after TSS.

Values in parentheses are International System of Units (SI) Normal range for IGF-1 (insulin-like growth factor 1) is 84 to 270 ng/mL (11-35.37 nmol/L). Normal range for GH (growth hormone) is less than 10 ng/mL or 10 µg/L.

## Treatment

The patient had a prolonged admission for heart failure at our institution requiring milrinone infusion. In the outpatient setting, he had been maintained on goal directed medical therapy (GDMT), including a beta-blocker, angiotensin receptor/neprilysin inhibitor, mineralocorticoid receptor antagonist, and a sodium-glucose co-transporter 2 inhibitor. After multidisciplinary transplant committee discussions, orthotopic heart transplant (OHT) was not offered due to concerns of transplant failure in the setting of active GH-secreting adenoma. In March 2022, he underwent LVAD placement, along with bioprosthetic aortic valve replacement, and an aneurysm repair. He tolerated the procedure well without any major postoperative complications and was continued on cabergoline 0.5 mg twice weekly. At our institution, pegvisomant was not considered for 2 reasons. There was concern that switching from lanreotide to pegvisomant could be associated with transient and occasional increase in tumor size due to loss of lanreotide-related shrinking (2). Furthermore, this therapy was no longer considered since he ultimately required treatment with a transplant.

The patient's IGF-1 level remained elevated at 528 ng/mL with a GH level of 4.2 ng/mL. Repeat MRI could not be completed due to the patient's LVAD. In October 2022, in concert with neurosurgery, otolaryngology (ENT), and cardiology collaboration, the patient underwent planned TSS for resection of his adenoma without complications. Prior to surgery, a complete pituitary panel showed GH 7.3 ng/mL (7.3 µg/L), prolactin 2.0 µg/L (86.96 pmol/L), TSH < 0.2 µU/mL, ACTH 58 pg/mL (12.76 pmol/L), FSH 4.3 mIU/mL (4.3 IU/L), and LH 4.7 mIU/mL (4.7 IU/L). Pathology showed pituitary adenoma with immunohistochemical staining positive for GH. Postoperatively, IGF-1 levels continued to decrease, reaching normal levels around month 4.

## Outcome and Follow-Up

**Figure 2.**
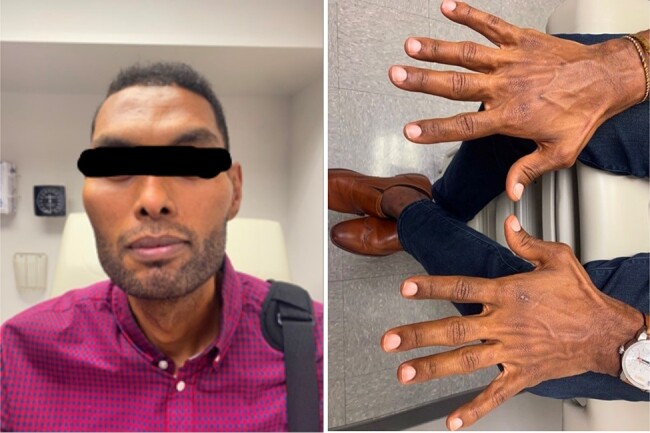
Seven months after TSS. Patient with coarse facial features and micrognathia on left. Notably, the patient felt reduced swelling in his fingers since TSS. Unfortunately, there are no photos from earlier in his disease course for comparison.

Since his TSS, the patient continues to be in remission, without need for pharmacologic intervention. He is doing well with his HeartMate3 LVAD (Abbott Cardiovascular, Minnesota, USA) and remains on the heart transplant wait list as a status 4 indication. When last seen in our endocrine clinic, the patient endorsed no complaints, and felt close to his baseline prior to the heart failure exacerbations. He noted that the swelling around his fingers was much improved (Fig. 2).

## Discussion

This patient's course highlights both an extremely rare but devastating consequence of undiagnosed acromegaly cardiomyopathy leading to congestive heart failure at time of presentation, followed by the deleterious effects of inability to maintain disease control with access to medicine and surgery, and how the role of LVADs may help patients with similar disease severity to be stabilized in the interim to a heart transplant.

Among the spectrum of cardiac manifestations due to acromegaly, there are 3 main categories: hypertension, cardiomyopathy, and arrythmias. While hypertension is the most common, with a prevalence of 22% at time of diagnosis, acromegaly-related cardiomyopathy resulting in overt systolic or diastolic heart failure is quite rare, effecting approximately 3% to 4% of cases, most likely since patients are typically diagnosed *prior* to the development of heart failure. As fibrosis slowly occurs, the heart undergoes 3 phases of injury. Phase one begins with biventricular hypertrophy with an initially hyperdynamic heart; medical treatment at this stage can reverse the damage. During the second phase, there is development of diastolic dysfunction and notably, patients can experience reduced exercise capacity. Oftentimes, patients are diagnosed at this stage, and if left untreated, will further progress to the final stage of overt congestive heart failure (3). At this point, patients may also have concurrent hypertension, valvular disease, and/or arrythmias, which can further worsen cardiomyopathy (4). A cohort study by Colao et al divided 25 patients with symptoms of acromegaly into 2 different categories based on disease duration (less than 5 years vs more than 5 years). It was shown that patients in the longer disease duration group had an increase in left ventricular mass and decrease in left ventricular function (5). Based on these reports, we can conjecture for our patient a similar story: he developed increasing phenotypic features of acromegaly in late adolescence, several years prior to presentation. Since then, he was likely undergoing years of cardiovascular fibrotic change, ultimately presenting with overt heart failure at age 37 years. In this patient's case, while his LVEF had originally improved on medical therapy (15% to 30%), the ultimate treatment, after removal of the GH-secreting adenoma, would be OHT. However, our patient was unable to obtain said transplant, as continued production of GH would damage the transplanted heart. LVAD was deemed the only option as the patient would require cardiac support from the device in order to be stable enough to pursue TSS and eventual transplant. One year later, the patient's acromegaly is in remission, and he is currently on the transplant waiting list. He follows regularly with the heart failure team for transplant status updates. In general, there are few documented cases of acromegalic patients with OHT, and even fewer with LVADs. While LVADs are commonly used as bridges to OHT in ischemic cardiomyopathy cases, this is relatively new for nonischemic cardiomyopathy in the setting of GH excess. One other similar case was noted by Reddy et al, in which LVAD provided stable transition. However, this case did not show any post-LVAD follow-up or provide information on the patient's history or objective data of his presentation and late disease control (6). While direct OHT was deemed unsafe in these patients, direct OHT prior to adenoma resection has been done. In a case by Dewanjee et al, the patient presented in cardiogenic shock, and despite treatment with milrinone, he ultimately required extracorporeal membrane oxygenation (ECMO) with transition to an Impella (Abiomed, Inc., Massachusetts, USA). Given the acuity of his presentation and despite “uncertainty of OHT in patient with untreated acromegaly,” the patient underwent OHT. While this was not an option for our patient, this case suggests that direct OHT could be a viable consideration in particular situations (7). However, even with successful OHT, irrespective of LVAD bridge, patients are still at risk of sudden death (8). These few cases demonstrate the inherent variability in treatment choice for cardiac life support in the setting of end-stage heart failure secondary to acromegaly.

Ultimately, this case exhibits a natural course of moderately increased autonomous GH secretion from a young age, not diagnosed until years later, and worsened by suboptimal treatment due to intermittent access to medical care. His journey highlights the common inequities of the American healthcare system. The medications' cost burden falls upon the patient and his access to insurance. However, the cost burden of the LVAD (a life-saving intervention) does not directly fall on the patient, making it accessible when urgently necessitated. This case emphasizes the importance of early detection and uninterrupted treatment, with hope for reversibility in heart function. However, in the few and unfortunate cases that progress to end-stage heart failure requiring advanced cardiac support, our patient demonstrates that the LVAD is a successful option as a bridge to eventual transplant, and we urge other institutions to consider this option.

## Learning Points

- Acromegaly-induced heart failure is one of the rarest cardiac complications, only diagnosed in patients who have been untreated long past their presenting physical manifestations.
- LVADs can be considered in patients with GH-induced heart failure with irreversible damage, necessitating heart transplant. They can be useful as a temporary measure of support, while the patient achieves medical or surgical control of the source of GH.
- Achieving biochemical control without delay is also imperative as a temporary measure while patient achieves medical or surgical control of the GH-secreting adenoma.

## Contributors

All authors made individual contributions to authorship. F.L.T. and M.K.S. were involved in the management of the patient and manuscript submission. M.M. was involved in manuscript writing. All authors reviewed and approved the final draft.

## Data Availability

Data sharing is not applicable to this article as no datasets were generated or analyzed during the current study.

## Abbreviations

ACTH: adrenocorticotropic hormone
EF: ejection fraction
FSH: follicle-stimulating hormone
GH: growth hormone
IGF-1: insulin-like growth factor 1
LH: luteinizing hormone
LVAD: left ventricular assist device
LVEF: left ventricular ejection fraction
MRI: magnetic resonance imaging
OHT: orthotopic heart transplant
TSH: thyrotropin (thyroid stimulating hormone)
TTE: transthoracic echocardiography
